# Clinical use of polygenic risk scores for detection of peripheral artery disease and cardiovascular events

**DOI:** 10.1371/journal.pone.0303610

**Published:** 2024-05-17

**Authors:** Jesutofunmi A. Omiye, Ilies Ghanzouri, Ivan Lopez, Fudi Wang, John Cabot, Saeed Amal, Jianqin Ye, Nicolas Gabriel Lopez, Faatihat Adebayo-Tijani, Elsie Gyang Ross

**Affiliations:** 1 Division of Vascular Surgery, Department of Surgery, Stanford University School of Medicine, Stanford, California, United States of America; 2 Center for Biomedical Informatics Research, Department of Medicine, Stanford University School of Medicine, Stanford, California, United States of America; 3 Department of Biomedical Data Science, Stanford University School of Medicine, Stanford, California, United States of America; 4 Department of Dermatology, Stanford University School of Medicine, Stanford, California, United States of America; 5 Department of Bioengineering, The Roux Institute at Northeastern University, Portland, Maine, United States of America; 6 Division of Vascular Surgery, Department of Surgery, UC San Diego School of Medicine, La Jolla, California, United States of America; Yale University School of Medicine, UNITED STATES

## Abstract

We have previously shown that polygenic risk scores (PRS) can improve risk stratification of peripheral artery disease (PAD) in a large, retrospective cohort. Here, we evaluate the potential of PRS in improving the detection of PAD and prediction of major adverse cardiovascular and cerebrovascular events (MACCE) and adverse events (AE) in an institutional patient cohort. We created a cohort of 278 patients (52 cases and 226 controls) and fit a PAD-specific PRS based on the weighted sum of risk alleles. We built traditional clinical risk models and machine learning (ML) models using clinical and genetic variables to detect PAD, MACCE, and AE. The models’ performances were measured using the area under the curve (AUC), net reclassification index (NRI), integrated discrimination improvement (IDI), and Brier score. We also evaluated the clinical utility of our PAD model using decision curve analysis (DCA). We found a modest, but not statistically significant improvement in the PAD detection model’s performance with the inclusion of PRS from 0.902 (95% CI: 0.846–0.957) (clinical variables only) to 0.909 (95% CI: 0.856–0.961) (clinical variables with PRS). The PRS inclusion significantly improved risk re-classification of PAD with an NRI of 0.07 (95% CI: 0.002–0.137), *p* = 0.04. For our ML model predicting MACCE, the addition of PRS did not significantly improve the AUC, however, NRI analysis demonstrated significant improvement in risk re-classification (*p* = 2e-05). Decision curve analysis showed higher net benefit of our combined PRS-clinical model across all thresholds of PAD detection. Including PRS to a clinical PAD-risk model was associated with improvement in risk stratification and clinical utility, although we did not see a significant change in AUC. This result underscores the potential clinical utility of incorporating PRS data into clinical risk models for prevalent PAD and the need for use of evaluation metrics that can discern the clinical impact of using new biomarkers in smaller populations.

## Introduction

Peripheral artery disease (PAD) is a ubiquitous condition of considerable morbidity and mortality worldwide. It is estimated that 12–20% of Americans over 60 years of age, and more than 230 million people globally are affected [1–3]. The economic burden of this condition is enormous with annual costs associated with PAD in the US projected to exceed $21 billion [4]. Atherosclerosis is rarely isolated to a single vascular bed and patients with PAD have a 3-to-6-fold increased risk of major adverse cardiovascular and cerebrovascular events (MACCE) [5–7].

Despite the severity of this condition, PAD often goes undiagnosed [8, 9]. Most patients do not receive a diagnosis until the late stages of the disease process when they are symptomatic. Therefore, PAD patients regularly require frequent and aggressive interventions to ensure amputation-free survival [10, 11]. Given the extensive treatment required for patients with end-stage disease, early detection could enable less costly lifestyle and pharmacologic interventions [9, 12, 13]. One way to achieve early detection is through the use of PAD risk scores. Although risk assessment tools in practice utilize clinical variables such as smoking and obesity, there has been a growing interest in exploring genetic information for the risk stratification of vascular diseases [14–18].

Genome-wide analysis studies (GWAS) using large repositories of genomic data have identified disease-specific genetic risk loci. In addition, the summary statistics from these studies can be used to create a polygenic risk score (PRS) which provides a summary assessment of an individual’s genetic risk of a condition [19–21]. We have previously created a PRS for PAD from the UK Biobank [22] and demonstrated that it can enhance the performance of a clinical risk model in a large retrospective cohort. We also showed that a PAD PRS was associated with the risk of other cardiovascular diseases such as coronary artery disease (CAD), congestive heart failure (CHF), and cerebrovascular disease (CVD) [22]. While PRS has been studied in large population cohorts, one of its barriers to implementation in the clinical setting is its practical integration with recognized risk factors [17]. Specifically, how well retrospective analysis of a large database translates to clinical practice in smaller patient cohorts remains unclear.

This study aims to address this gap and expand on our earlier work by exploring the clinical utility of a PRS in detecting PAD risk and predicting MACCE and adverse events (AE) using data from our own institution. In doing so, we aim to use data science research to understand how genetic data can potentially be used to alter real world practice and improve patient care.

## Materials and methods

### Study cohort

This study was approved by Stanford University’s institutional review board (IRB 6208). The genetic data for this study were obtained from the Stanford Precision Health Biobank [23], a collection of blood samples from patients that can be used for medical research. The biobank started recruiting patients in 2014 and we pulled data up until 2019. At the time of our analysis, approximately 3,150 patients had their data and biobank samples available for analysis, with a male to female ratio of 1.1 to 1, and informed written consent was obtained from all participants to have their samples and clinical data analyzed for research purposes. The data for our study was accessed on April 1, 2022.

To create our study cohort, we used the Phenotype KnowledgeBase (PheKB) [24] cohort definition for PAD to identify cases of PAD with a Biobank sample and matched this to a random 10% age-matched sample of controls with a Biobank sample but no PAD diagnosis. Sex matching was not done due to known PAD epidemiology, where men and women are affected equally [25]. A manual chart review tool was used to confirm PAD diagnosis and control status. Patients were classified as having PAD on chart review if they had a diagnosis of PAD in their medical records, an ankle brachial index (ABI) of less than 0.9 on either leg, a history of revascularization surgery, and/or documentation of diminished or absent pedal pulses or wounds attributable to arterial disease. They were excluded if they had an ABI greater than 1.4 or non-compressible tibial vessels. Controls were defined as those without a diagnosis of PAD, no history of PAD-related procedures, and/or an ABI within normal limits. The codes for defining the PAD cohort and controls are available in S1 File.

Electronic Health Record (EHR) data were retrieved from the STAnford medicine Research data Repository—Observational Medical Outcomes Partnership (STARR-OMOP) [26]. This database represents a de-identified research version of our EHR data for over 3 million patients from Stanford hospitals and clinics. It is maintained by the Stanford Medicine Research IT team. The Stanford Research IT team linked the EHR data with the genetic data and none of the authors had access to identifying information of the study participants.

### Genetic analysis

#### Genotype quality control and imputation

Blood samples were collected from the Precision Health Biobank, and DNA was extracted using standard techniques. All samples were genotyped using the Axiom Precision Medicine Diversity Array. Individuals with a call rate of less than 98% were excluded to control for genotype quality. We used PLINK 1.9 to exclude individuals with more than 5% missing data, gender mismatch with typed X-linked markers, or excess autosomal heterozygosity (>0.33). Additionally, duplicates and individuals identified as first-degree relatives were removed using IBS probabilities. We also excluded single nucleotide polymorphisms (SNPs) with more than 2% missing data, with a minor allele frequency (MAF) below 1%, and those that failed the test for Hardy-Weinberg equilibrium (*p* < 1×10^−5^). After the application of a quality-control criteria, there were 875,265 remaining SNPs from our cohort to be analyzed.

Genotype data were then phased using SHAPEIT4.2 and imputed using IMPUTE5 with variant positions from the 1000 Genomes Phase 3 data [27]. SNPs with MAF below 1%, imputation quality score below 0.5, or a p-value of deviation from Hardy-Weinberg equilibrium less than 1×10^−5^ were excluded in further analysis. Finally, a total of 18,349,371 imputed SNPs passed quality control.

#### SNPs and polygenic risk score

**Fig 1:**
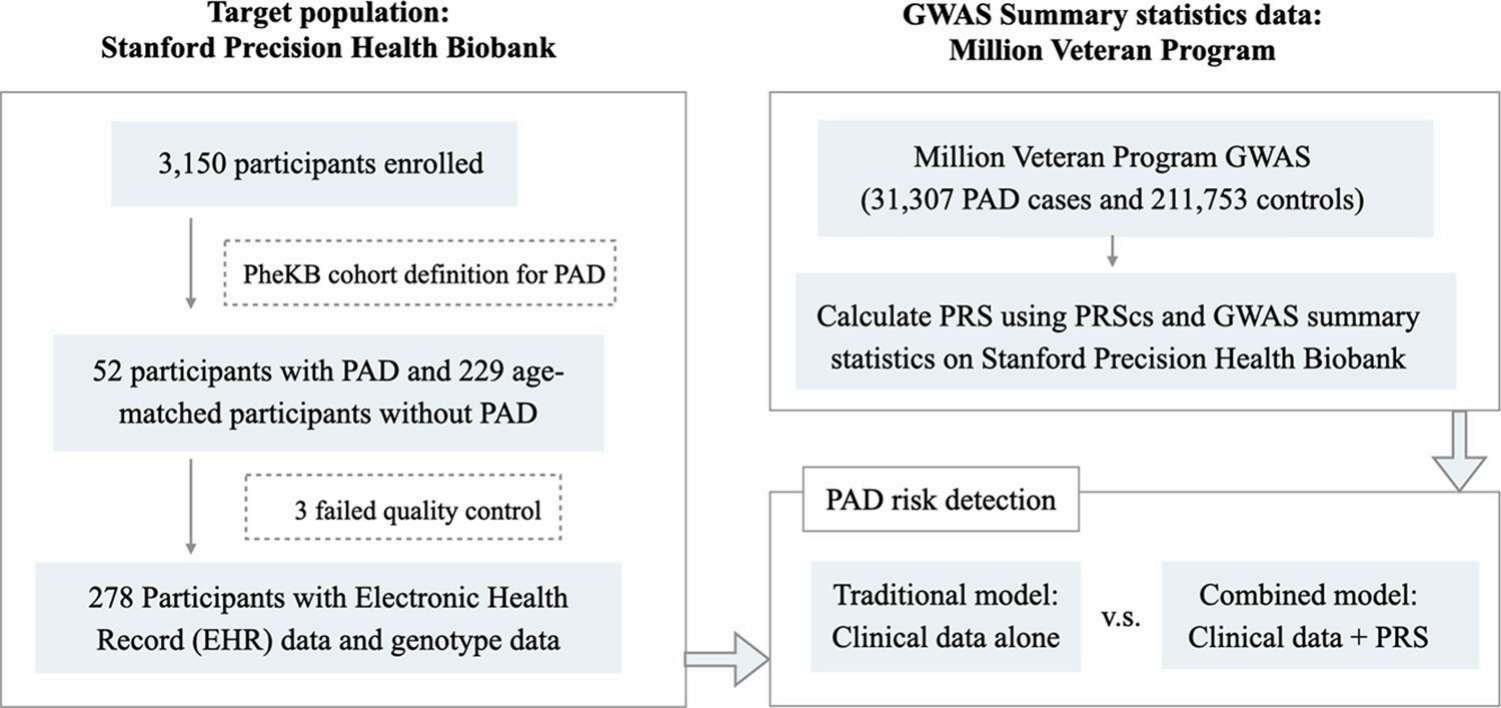
Overview of the study. This flowchart shows an overview of the methodology used in developing the PRS and models.

We derived a PAD polygenic risk score (PRS) based on the weighted sum of risk alleles using summary statistics of PAD from the Million Veteran Program (MVP) [28]. The MVP GWAS analysis consisted of 31,307 individuals with PAD and 211,753 PAD-free controls. We calculated PRS using PRScs which applied a Bayesian Shrinkage parameter on effect size estimates for each SNP based on GWAS summary statistics [29]. In order to analyze the contribution of any individual SNP, we used summary statistics from both MVP and the UK Biobank [30] and used a cut-off of 5e-8 resulting in 54 top SNPs associated with PAD. Lastly, we removed 12 SNPs that did not exist in our patient cohort. An overview of the methodology is shown in Fig 1.

### Model development

#### Detecting PAD

We created a logistic regression model to detect PAD risk using clinical and genetic data. This involved developing 2 separate models for disease detection—one using clinical data alone and another combining clinical data with the PRS—for a final PAD risk detection model.

#### Clinical model

Using patients’ EHR data, we adapted a previously published PAD risk score to calculate individual-level risk of PAD [31]. Clinical risk factors were extracted from patients’ data as previously described [32] and included a history of cerebrovascular accident (CVA), coronary artery disease (CAD), heart failure (CHF), hypertension (HTN), diabetes (DM), hyperlipidemia (HLD), body mass index (BMI), and smoking status. Demographic factors like age, sex, and ethnic group were also included.

#### Combined data model

Features of the combined model included the derived patient-level PRS adjusted for age, sex assigned at birth, and genetic ancestry, alongside the clinical and demographic variables described above. Using generalized linear modeling we evaluated the model performance with PRS as a continuous variable as well as a binary variable by testing threshold values. A threshold PRS of greater than 0.18 was classified as a high PRS for our model. This threshold was established based on its optimal performance in an independent linear classification analysis, where PRS was the only feature.

### Predicting patient outcomes

There were two major patient outcomes of interest, MACCE, and AE. Our aim was to evaluate whether, and to what extent, genetic data influence model accuracy for predicting these outcomes. MACCE was defined as the presence of an ICD-10, CPT, or SNOMED code related to a cerebrovascular or cardiovascular event, and adverse events (AE) were defined as the combination of MACCE and death or amputation (S1 File). If patients had multiple MACCE or AE, the earliest event was chosen for inclusion. To predict these outcomes, we used age, sex, genetic ancestry, clinical risk factors from our clinical model, and genetic data in the form of PRS and individual SNPs as features. The motivation for evaluating individual SNPs as variables was due to our interest in evaluating to what extent known SNP associations with cardiovascular disease identified in population studies could help predict adverse outcomes on an individual basis.

We used the Random Forest (RF) algorithms [33] to build our models for MACCE and AE prediction. This was chosen for its ability to handle large numbers of features with complex nonlinear relationships, making it well-suited for analyzing data of different categories i.e. genetic and clinical data. Furthermore, RF algorithms have a feature importance component that enables understanding which features contribute most to a model’s predictive capacity.

Models were developed with clinical data alone and clinical data plus either PRS or individual SNPs. Ten-fold cross-validation was used, and the data were divided into a 70/30 train and test set with model performance evaluated on the test set.

#### Performance measures

The discriminative power of each model was evaluated using the area under the curve (AUC). We also used Delong’s test of significance to compare the AUC of the clinical and combined (clinical plus PRS/clinical plus SNPs) models for PAD risk prediction. Because AUC can be a crude assessment of the value of adding a genetic marker to a risk prediction model, we extended our performance assessment to include net reclassification index (NRI) and integrated discrimination improvement (IDI) [34]. NRI helps quantify the net change in correctly reclassifying patients across defined risk groups and is often used for measuring the prediction improvement of new biomarkers [35]. IDI measures the overall improvement in model discrimination [36]. Since CVDs are clinically managed based on risk categories that patients fall into, it was imperative to evaluate how our model performs on these risk classification tasks. For the NRI analysis of the PAD detection model, we used the thresholds of <5% for low risk, 5-<25%, and ≥25% for intermediate and high risk respectively. For the outcome models, we used the Framingham risk categories [37], and applied the following stratification: <10%, 10- <20%, and ≥20% for low, intermediate, and high risk categories. We report the categorical NRI, associated IDI, and continuous NRI. In addition, we calculated the Brier score [38, 39] for both the clinical and clinical plus genetic models to compare calibration performance and accuracy. The lower the Brier score, the better for both calibration and discrimination.

While the above metrics give a sense of model discrimination and calibration, they do not fully capture how useful a model could be if implemented in the clinical setting. Decision curve analysis (DCA) is a method used to evaluate the clinical usefulness of prediction models by comparing their net benefits with default strategies of treating all or no patients [40]. DCA can be used to estimate the added value of models or tests, and can help determine their potential impact in clinical practice [41]. The net benefit is calculated across a range of threshold probabilities, which is defined as the minimum probability of disease at which intervention becomes appropriate [42]. It complements the AUC as it includes the consequence of decisions made based on the model’s output.

We used R, version 3.6.3 (R Project for Statistical Computing) [43], Python, version 3.7.10 [44], and scikit-learn library (version 1.0.2) [45] for building our model, testing, and statistical analysis. We used 2-sided tests with *p* < 0.05 as our threshold for statistical significance.

## Results

### Study cohort characteristics

A total of 278 patients were included in the final cohort. Of these patients, 52 had PAD. There was no significant difference in age between groups, which was expected since we performed age-matching. PAD patients had a higher proportion of males compared to control (82.7% vs 50%, *p* = 2.811x10^-5^). As expected, those with PAD had a higher prevalence of diabetes than non-PAD patients (40.4% vs 13.2%, *p* = 1.327x10^-5^) and a higher prevalence of CAD (96.2% vs 69%, *p* = 1.178x10^-4^). As expected, MACCE and AE occurred more frequently in those with PAD (73.08% and 80.77%, respectively) compared to non-PAD patients (32.3% and 36.3%, respectively). Table 1 provides a summary of a comparison of baseline characteristics between cases and controls.

**Table 1:** Baseline characteristics of our study cohort.

Variable	No PAD (n = 226)	PAD (n = 52)	*p*-Value*
**Age, mean (SD), years**	70.4 (9.9)	68.4 (9.5)	0.19
**Sex, male (%)**	112 (49.6)	43 (82.7)	2.81x10^-5^
**Genetic ancestry, Caucasian (%)**	208 (92.0)	42 (80.8)	0.06
**BMI, mean (SD)**	27.5 (5.6)	27.3(3.8)	0.75
**Diabetes, n (%)**	30 (13.3)	21 (40.4)	1.33x10^-5^
**Hyperlipidemia, n (%)**	83 (36.7)	27 (51.9)	0.06
**Hypertension, n (%)**	5 (2.2)	3 (5.8)	0.36
**Current Smoker, n (%)**	57 (25.2)	10 (19.2)	0.46
**Coronary artery disease, n (%)**	156 (69.0)	50 (96.2)	1.18x10^-4^
**Heart Failure, n (%)**	136 (60.2)	43 (82.7)	3.8x10^-3^
**Cerebrovascular disease, n (%)**	179 (79.2)	49 (94.2)	1.91x10^-2^
**MACCE, n (%)**	73 (32.3)	38 (73.1)	1.47x10^-7^
**AE, n (%)**	82 (36.3)	42 (80.8)	1.48x10^-8^

*Student’s t-test used for continuous variables and chi-squared test for categorical.

### Results of PAD prediction models

**Fig 2:**
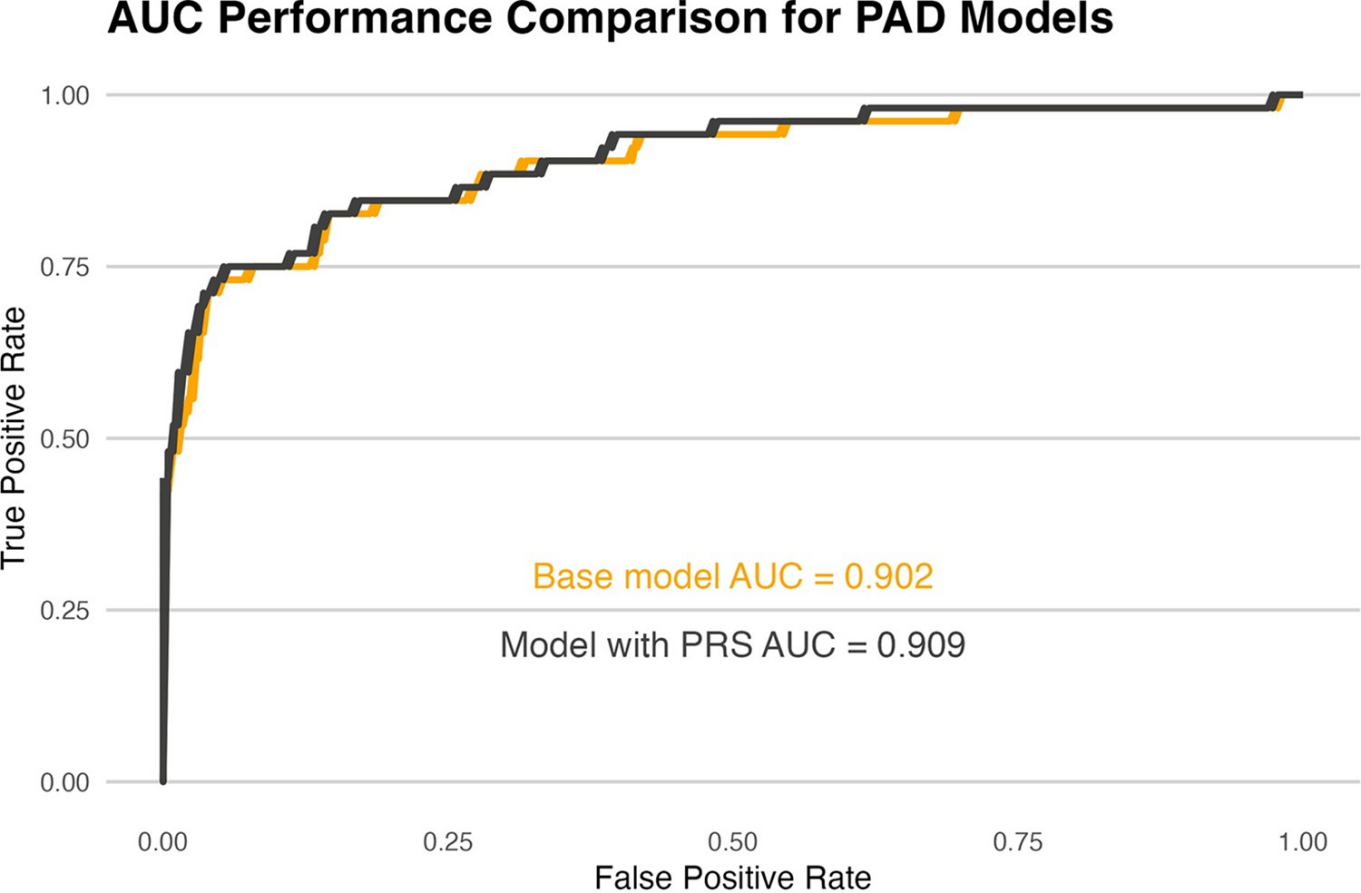
ROC curve for PAD detection model. ROC curve comparing the clinical-only and combined-PRS model showing a slightly larger AUC with the PRS model.

Our model with clinical data alone achieved an AUC of 0.902 (95% CI: 0.846–0.957). Adding a PAD-specific PRS to this model increased the AUC to 0.909 (95% CI: 0.856–0.961), though this was not statistically significant (*p =* 0.18) (Fig 2). The combined model showed an overall categorical-NRI of 0.069 (95% CI: 0.002–0.137) compared to the clinical model, which was statistically significant (*p* = 0.04). The patients in the intermediate risk group had the highest reclassification with the addition of the PRS (Table 2).

**Table 2:** NRI Result for the clinical (initial) and combined PAD detection models.

Combined Model With PRS					
Initial Model	Low Risk (<5%)		Intermediate Risk (5–25%)	High Risk (>25%)	Percent Reclassified
**Low Risk (0–5%)**	99		9	0	9%
**Intermediate Risk (5–25%)**	14		83	2	16%
**High Risk (>25%)**	0		2	69	3%
Net Reclassification Index					0.0694
*p* Value					0.044

**Fig 3:**
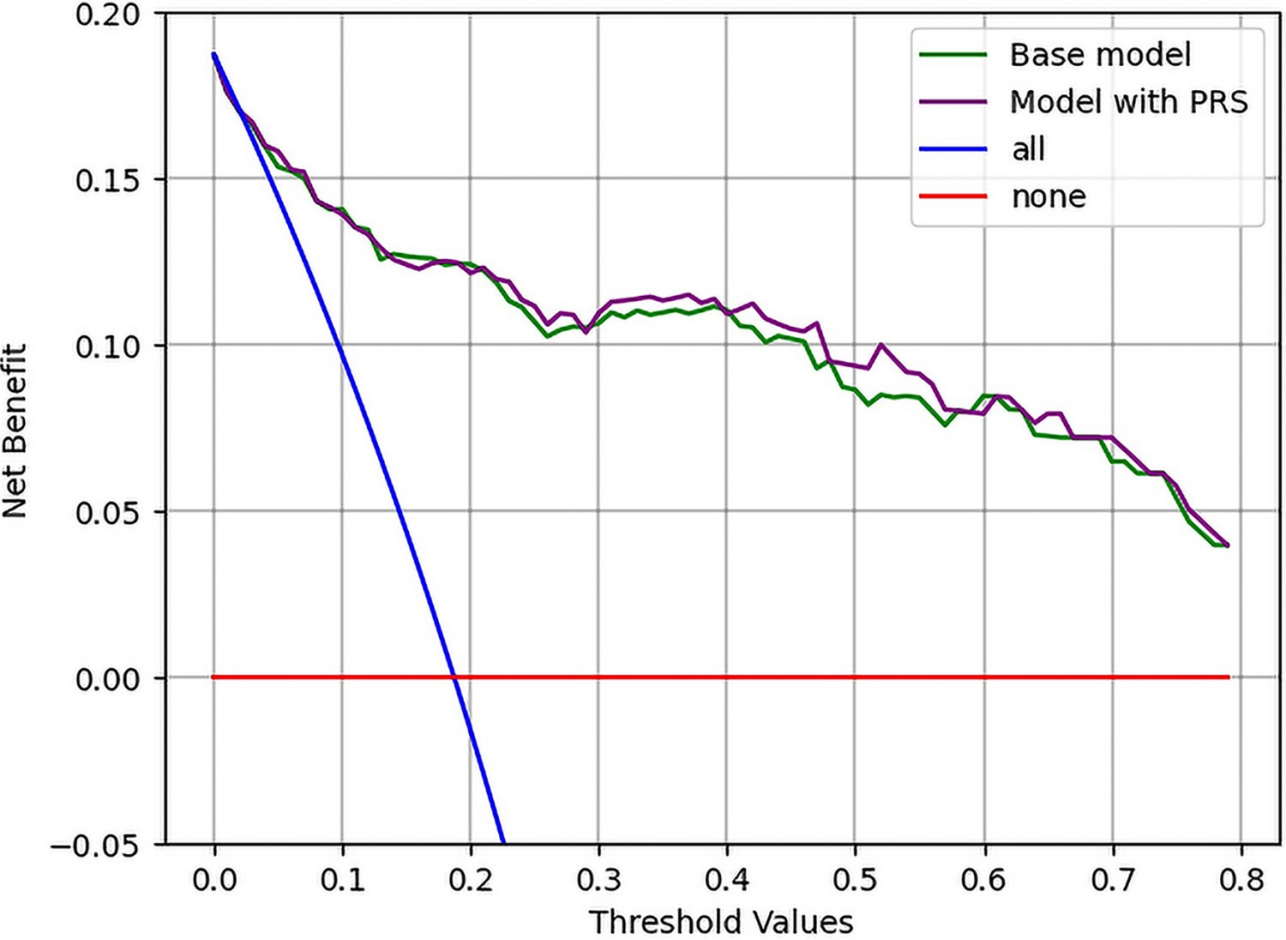
Decision curve analysis plot for the two models. The vertical axis shows the net benefit for using the models and the horizontal axis displays the threshold probability values for detecting PAD. Screen all is shown by the blue line which indicates a case in which all patients that present will be screened, while the screen none line shows the alternate scenario. We can see that the purple line illustrating the combined model with PRS dominates the clinical model across all the thresholds. Therefore, the preferred model would be the model with PRS.

Specifically, 14 of the 99 patients initially classified as intermediate group in the clinical model were reclassified to the low risk group, and 2 patients were reclassified to the high risk category based on the addition of the PRS. The Positive NRI demonstrates that the combined model correctly moves more individuals with PAD to higher risk categories and those without PAD to lower risk categories [36]. A complete analysis of the NRI is shown in (S1 File). The IDI showed a significant improvement in discrimination of 0.0092 (95% CI: 0.0041–0.0143) with *p* = 0.00046, when adding the PRS variable. The Brier score was 0.172 for the clinical model and it reduced to 0.169 for the combined model, demonstrating improved accuracy of the combined PRS model. For our DCA, we illustrate 4 clinical strategies (Fig 3) to detect PAD—screen all patients for PAD (blue), screen no patients for PAD (red), and screen based on our clinical-only model (green), or screen based on our combined clinical and genetic model (purple). Our combined PRS model offers the most net benefit over the other strategies across a wide threshold of PAD risk thresholds.

### Results of outcome detection models

There were a total of 38 patients (73%) with PAD who experienced MACCE while 73 non-PAD patients (32%) experienced MACCE. The average time to MACCE after PAD diagnosis was 6.2 months. For those without PAD, the average time to MACCE after entry into the healthcare system was 31.2 months. The most common event was a myocardial infarction for both PAD and non-PAD patients.

For MACCE prediction, our RF model showed high discrimination using clinical data alone with an AUC of 0.830 (95% CI: 0.747–0.914). Adding the PRS did not improve performance (AUC 0.818, 95% CI: 0.727–0.910, *p* = 0.436). For the NRI, adding PRS to our clinical data only model resulted in the reclassification of 9 out of 14 individuals from the intermediate risk category into the lower risk category, a reclassification rate of 64%, and 5 of 68 individuals from a higher risk category into intermediate and low risk groups, for a reclassification rate of 7%. These reclassification rates resulted in an overall continuous NRI of 0.852 (95% CI: 0.462–1.242, *p* = 2e-05) (Table 3). The IDI was 0.083 (95% CI: 0.046–0.119, *p <* 0.05) supporting a significantly improved discrimination of the combined PRS model. The Brier score for the model with PRS was also lower (better) at 0.178 compared to 0.192. However, using individual SNPs instead of the PRS variable showed overall poorer model discriminatory performance with an AUC of 0.802 (95% CI: 0.708–0.896).

**Table 3:** Net reclassification improvement results for the MACCE prediction model.

Combined Model With PRS (For MACCE Outcome)				
Initial Model	Low Risk (<10%)	Intermediate Risk (10–20%)	High Risk (>20%)	Percent Reclassified
**Low Risk (<10%)**	2	0	0	0%
**Intermediate Risk (10–20%)**	9	5	0	64%
**High Risk (>20%)**	1	4	63	7%
Net Reclassification Index				0.8522
*p* Value				2e-05

**Fig 4:**
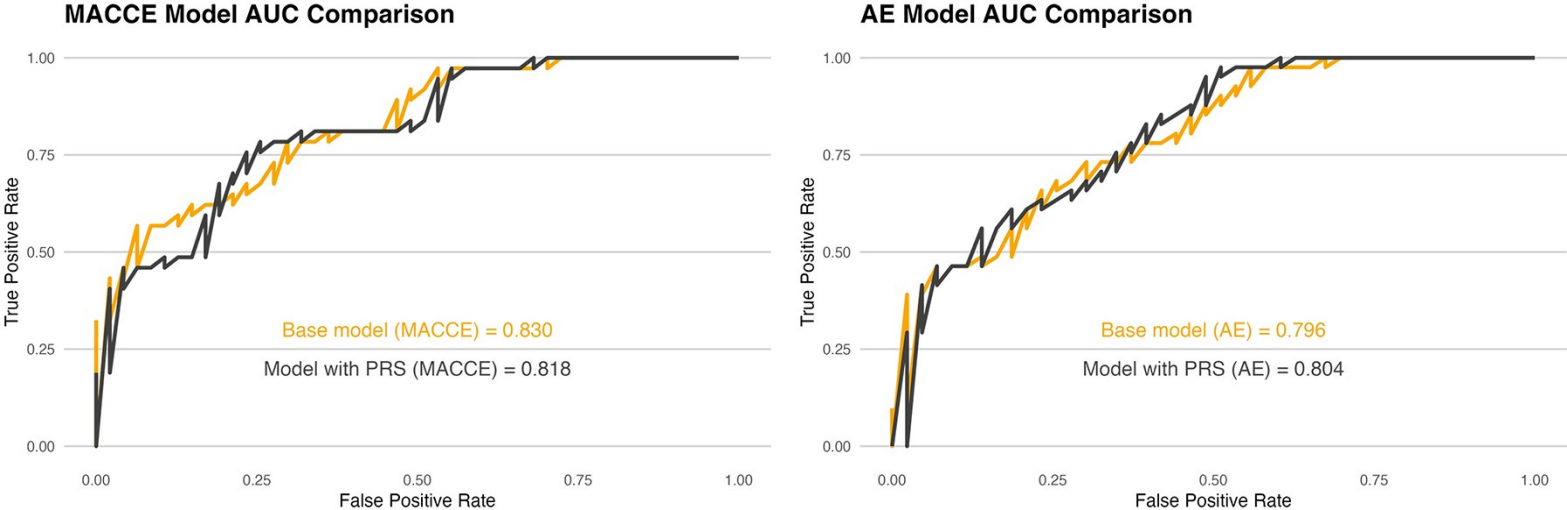
ROC plots for the RF models for MACCE (left) and AE (right), with and without PRS.

AE occurred in 42 (81%) PAD patients and 82 (36%) patients without PAD. The RF model with clinical data showed an AUC of 0.796 (95% CI:0.705–0.888), while the combined PRS model increased discrimination to an AUC of 0.804 (95% CI:0.709–0.898, *p* = 0.531) (Fig 4). Including the PRS for the NRI analysis, showed reclassification for 2 of 3 patients from the low risk group to the intermediate group for a reclassification rate of 67%. Also, 1 of 11 patients was reclassified from the intermediate category to the high risk group. The overall continuous NRI was worse with the combined PRS model at -0.2779 (95% CI -0.696–0.1402, *p =* 0.19261). The IDI metric was -0.0103 (95% CI -0.024–0.004, *p =* 0.15) although not significant. Brier scores for both models were 0.189, indicating that adding the PRS to the AE clinical prediction model did not result in significant improvement in model performance.

**Fig 5:**
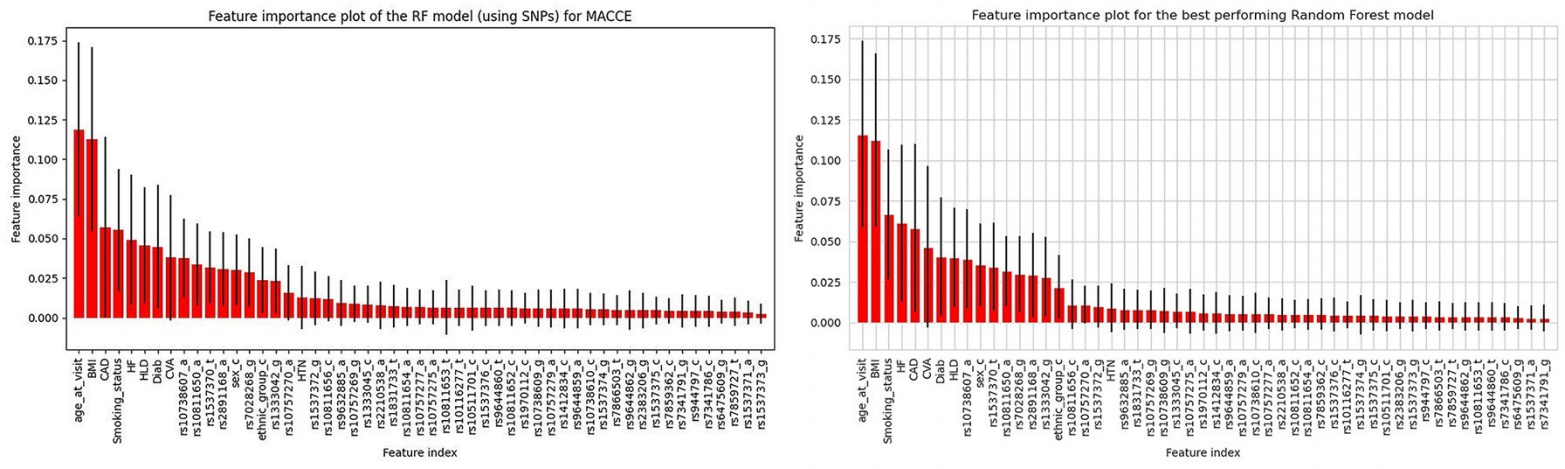
Feature importance plots for the RF models (using SNP data) to predict MACCE (left) and AE (right). We see that both models prioritize very similar genetic information, alongside the well-known clinical variables.

Using individual SNPs instead of PRS improved model AUC—0.818 (95% CI: 0.733–0.904) compared to 0.793 (95% CI: 0.699–0.887) without SNPs, however this was not significant. Finally, feature importance evaluation of the models utilizing SNPs to predict both MACCE and AE revealed that age was the most impactful feature and the most informative SNPs were *rs10738607_a*, *rs1537370_t* and *rs10811650_a* (Fig 5).

## Discussion

In this study, we examined the utility of genetic data for improving PAD detection and MACCE and AE prediction. We found that even in a small sample size adding a PAD-specific PRS to a clinical PAD detection model can better refine patient risk stratification, as evidenced by improvement in NRI and IDI. Although AUC slightly improved in each model, these changes were not significant. Furthermore, we found that with these small improvements in net reclassification and discrimination, a strategy of using clinical and genetic data to identify patients who should be screened for PAD may increase net utility compared to using clinical data alone.

There is a growing interest in the use of genetic data for personalized disease risk stratification and prediction. Increasingly, these data are utilized in the form of a PRS. Chaudhury et al. showed a PRS-augmented risk model successfully predicted conversion from mild cognitive impairment to late-onset Alzheimer’s disease at an accuracy of 85.2% [46]. In the realm of cardiovascular disease, the majority of studies have examined the utility of PRS to improve risk stratification for coronary artery disease (CAD) and have thus far demonstrated mixed results. For example, Inouye and colleagues showed that their PRS constructed from 1.7 million genetic variants significantly improved CAD risk stratification with individuals in the top 20% of risk compared to those in the bottom 20% [47]. Khera et al, using UK Biobank data, also showed that there was a role for PRS in the risk assessment of CAD [19].

On the other hand, Elliott and colleagues [16] found that in a large observational cohort, also using data from UK Biobank participants, while PRS combined with clinical data resulted in a statistically significant difference in model performance, clinically, there was only a modest improvement in risk stratification. Specifically, they found that adding PRS to the pooled cohort equations only improved stratification for a small cohort of individuals. Marston and colleagues also showed that PRS was helpful in risk stratification for CAD, although the majority of the benefit was seen in the younger patient population [17]. This makes sense as the traditional clinical risk factors may take time to develop, while genetic predispositions are carried throughout life.

Our findings of statistically significant patient risk restratification evidenced by both NRI and IDI for PAD and MACCE detection is notable, and aligns with previous literature on CAD. However, it is worthy of note that the slight change in AUC was not statistically significant. This could be due to our small sample size and limitations of AUC in evaluating the added value of a biomarker [36]. NRI, IDI, and the DCA indicate that there is some information in the PRS to help adjust patient risk factors, and if done at scale, this observed small difference could lead to larger differences in population health outcomes.

In addition to detecting prevalent disease, genetic data can potentially be used for predicting the risk of future adverse outcomes. For AE, overall model performance for our clinical model was high and PRS did not significantly improve upon this. Interestingly though, when we evaluated feature importance of specific SNPs, we saw that *rs10738607_a* and *rs1537370_t*, were highly influential in AE and MACCE prediction. These findings are consistent with literature. For example, SNP *rs10738607_a* has been associated with high cholesterol levels [48], and, its variant, *rs10738607_g*, has been closely linked with coronary artery disease and myocardial infarction in some studies [49, 50]. Also, *rs1537370_t*, has been closely linked with coronary artery calcification and implicated in myocardial infarction [51]. It is possible that if patients are genotyped at younger ages and followed for longer periods of time, these variants can prove to be more predictive of outcomes when combined with clinical and behavioral data.

While these results are of great academic and scientific interest, the question of real-world clinical applicability lingers. The barriers to entry of using genetic data in everyday clinical practice are two-fold. First, statistical significance does not necessarily confer clinical significance and thus meaningful clinical value must be demonstrated. Using decision curve analysis, we found that the combined (PRS plus clinical model) improved net benefit, though modestly, compared to using clinical data alone for PAD screening. Second, the overall cost of genome sequencing may still be considered to be prohibitive. However, if identifying the results could lead to halting development of disease or preventing more severe sequelae, conducting the genetic sequencing for younger patients may ultimately save on healthcare costs in the long run. For example, while genetic testing expenses can fluctuate considerably, the cost per megabase (Mb) averaged $0.006 in August 2021, resulting in an approximate cost of $562 for sequencing a genome [52]. The average annual clinical expense per individual with PAD was estimated to be $11,553 between 2011 and 2014 [53]. There is a potential to save money by catching patients early and reducing high health care costs such as surgical procedures. Compared to no implementation of genetic and clinical-based model screening, the economics of model-detected PAD could be favorable and can improve clinicians’ ability to find patients at risk.

While novel, our study has some limitations. Our biobank is made up of a small cohort of volunteers from the patients treated at one hospital. This population includes a significant proportion of individuals of higher socioeconomic status and of European descent. These characteristics may limit the generalizability of our models to regions with different genetic diversity and socioeconomic factors [54]. However, our goal was not to build a generalizable model but to show the possibility of using local patients’ genetic and EHR data for building risk models. Furthermore, the size of our biobank is relatively small compared to other biobanks like the UK Biobank thus underpowering our study. Despite these shortcomings, we opted to use our biobank as the granularity of data available allowed for the novel combination EHR and genetic data. And although small, we found some important differences across the models. Our observed model improvements are modest and should spark further evaluation of how best to build precision health models for local implementation. Lastly, there is the potential for bias in PRS estimations and decisions on how to generate a PRS—pruning and thresholding versus Bayesian models could produce different results. However, as detailed in the statistical fine mapping literature, uncertainty in PRS estimation decreases with the sample size of the GWAS training data [55]. In our case, we used a large GWAS study to develop our PRS, and we evaluated both pruning and thresholding, and Bayesian methods and found that these methods did not produce significant result differences. Impactful futures studies should focus on prospectively evaluating the clinical utility of adding genetic risk scores to clinical models for PAD detection and risk prediction, and evaluating how models developed in larger datasets compare to models built on local data alone.

## Conclusion

The findings of this study show that in a small cohort of patients, combining clinical data with a PAD PRS shows some promise in refining risk stratification for PAD detection and MACCE prediction. There is also the potential to gain clinical utility with this approach. Future work should focus on optimizing ways to build and implement precision-health screening for PAD in a more generalizable fashion and prospectively evaluating the clinical impact.

## Supporting information

S1 File This contains the codes for identifying PAD, MACCE, AE, and other supplementary results. (PDF)

S2 File This contains the SNP data weights used for our study. (ZIP)
