# Supplementary material for: Clinical use of polygenic risk scores for detection of peripheral artery disease and cardiovascular events
Source: PLoS One. 2024 May 17;19(5):e0303610. doi: 10.1371/journal.pone.0303610 (PMC11101066; doi:10.1371/journal.pone.0303610)
Supplement: S1 File — (PDF) [file pone.0303610.s001.pdf]

SUPPLEMENTARY INFORMATION:

S1 Text: Algorithm/Code for identifying PAD Cases and Controls for RIC Chart Review Tool

PAD CASES:

Any of these codes

ICD9: 440.2*, 440.3*, 440.8, 440.4, 443.9
ICD-10: I70.2*,I70.3*,I70.4*,I70.5*,I70.6*,I70.7*,I70.8,I70.92,I73.9

OR any of these procedures

ICD-9-CM: 39.50, 39.90, 38.18

ICD-10-PCS: 047C3*, 047D3*, 047H3*, 047J3*, 047K3*, 047L3*, 047M3*, 047N3*, 047P3*, 047Q3*, 047R3*, 047S3*, 047T3*, 047U3*, 047V3*, 047W3*, 047Y3*, 04C00ZZ, 04C03ZZ, 04CC0Z*, 04CC3Z*, 04CC4Z*, 04CD*, 04CH*, 04CJ*, 04CK*, 04CK*, 04CL*, 04CM*, 04CN*, 04CP*, 04CQ*, 04CR*, 04CS*, 04CT*, 04CU*, 04CV*, 04CY*, 04100J7, 04100J8, 04100JF, 04100JG, 04100JH, 04100JJ, 04100JK, 041C0JD, 041C0JH, 041D0JF, 041D0JJ, 041H0JH, 041H0KJ, 041H4JH, 041J0JJ, 041J4JJ, 041K0J*, 041K0Z*, 041L09*, 041L0J*, 041L0K*, 041L0Z*, 041M*, 041N*, 041S0JQ, 041U0AP

CPT code: 37205, 37206, 37207, 37208, 37236, 37237, 37184, 37185, 37186, 35302, 35303, 35304, 35305, 35306, 35331, 35351, 35355, 35361, 35363, 35371, 35372, 35381, 35452, 35454, 35456, 35459, 35470, 35472, 35473, 35474, 35483, 35492, 35493, 35495, 35521, 35533, 35537, 35538, 35539, 35540, 35556, 35558, 35563, 35565, 35566, 35571, 35583, 35585, 35587, 35621, 35623, 35637, 35638, 35646, 35647, 35654, 35656, 35661, 35663, 35665, 35666, 35671, 35700, 35876, 35879, 35881, 35883, 35884, 37184, 37185, 37186, 37205, 37206, 37207, 37208, 0236T, 0237T, 0238T, 37225, 37224, 37227, 37226, 37222, 37223, 37220, 37221, 37229, 37228, 37231, 37230, 37233, 37232, 37235, 37234, 35548

Without these diagnosis codes in the record

ICD-9: 736.3*, 736.4*, 736.5, 736.6, 736.7*, 736.8*, 736.9, 735.*, 754.3*, 754.4*, 754.5*, 754.6*, 754.7*, 755.02, 755.13, 755.14, 755.3, 755.4, 755.6*, 755.8, 759.7, 759.89, 895.*, 896.*, 897.*, 820.*, 821.*, 822.*, 823.*, 824.*, 825.*, 826.*, 827.*, 828.*, 829.*, 835.*, 836.*, 837.*, 838.*, 904.*, 928.*, 929.*, 959.6, 959.7, 996.4*, 996.66, 996.67, 996.77, 996.78

ICD-10: M21.05*, M21.06*, M21.07*, M21.15*, M21.16*, M21.17*, M21.7*, M21.869, M21.6X*, M21.85*, M21.86*, M21.95*, M21.96*, M20.1*, M20.2*, M20.3*, M20.*, M20.5*, M20.6*, Q65.**, Q68.1*, Q68.2*, Q68.3*, Q68.4*, Q68.5*, Q66.3*, Q66.4*, Q66.5*, Q66.6*, Q66.7*, Q66.8*, Q69.2*, Q70.2*, Q70.3*, Q72.**, Q74.8, Q74.9, Q89.7, E78.71, E78.72,

Q87.2, Q87.3, Q87.5, Q87.81, Q87.82, Q87.89, Q89.8, S72.**, S82.**, S92.**, T14.8*, M24.35*, M24.36*, M24.37*, S83.**, S93.**, S75.**, S79.**, S99.**, S77.**, S89.**, T84.**

OR any of these procedures

ICD-9-CM: 84.1*

ICD-10-PCS: 0Y6*

CPT: 27590, 27591 ,27592, 27598, 27888, 27889, 28800, 28805

Without these diagnosis codes in the record
ICD-9: 170.6, 170.7, 170.8, 170.9, 171.3, 172.7, 173.7, 198.5, 344.1, 711.0, 728.86, 733.2, 736.3*, 736.4*, 736.5, 736.6, 736.7*, 736.8*, 736.9, 735.**, 754.3*,754.4*,754.5*,754.6*,754.7*,755.02, 755.13, 755.14, 755.3, 755.4, 755.6*, 755.8, 759.7, 759.89, 820.*, 821.*, 822.*, 823.*, 824.*, 825.*, 826.*, 827.*, 828.*, 829.**, 835.*, 836.*, 837.*, 838.*, 890.*, 891, 895.*, 896.*, 897.*, 904.*, 905.4, 928.*, 929.*,959.6, 959.7,996.4×, 996.66, 996.67, 996.77, 996.78.

ICD-10: C41.4, C40.2*, C40.3*, C41.9, C49.2*,C43.7*, D03.7*, C44.7*, C79.51, C79.52, G82.*, M00.*, M72.0, M85.65*, M85.66*, M85.67*, M85.68, M85.69, Z89.6*, M21.05*, M21.06*, M21.07*, M21.15*, M21.16*, M21.17*, M21.7*, M21.869, M21.6X*, M21.85*, M21.86*, M21.95*, M21.96*, M20.1*, M20.2*, M20.3*, M20.*, M20.5*, M20.6*, Q65.*, Q68.1*, Q68.2*, Q68.3*, Q68.4*, Q68.5*, Q66.3*, Q66.4*, Q66.5*, Q66.6*, Q66.7*, Q66.8*, Q69.2*, Q70.2*, Q70.3*, Q72.**, Q74.8, Q74.9, Q89.7, E78.71, E78.72, Q87.2, Q87.3, Q87.5, Q87.81, Q87.82, Q87.89, Q89.8, S72.*, S82.*, S92.*, T14.8*, M24.35*, M24.36*, M24.37*, S83.*, S93.*, S71.109A, S71.009A, S81.009A, S81.809A, S91.009A, S98.*, S88.*, S75.*, M84.*, S79.*, S99.*, S77.*, S89.*, T84.*

PAD CONTROLS:

Those without the following diagnosis codes:

ICD9: 440.2*, 440.3*, 440.8, 440.4, 443.9
ICD10: I70.2*,I70.3*,I70.4*,I70.5*,I70.6*,I70.7*,I70.8,I70.92,I73.9

And without the following Procedure codes:

ICD-9-CM: 39.50, 39.90, 38.18, 84.1*

ICD-10-PCS: 047C3*, 047D3*, 047H3*, 047J3*, 047K3*, 047L3*, 047M3*, 047N3*, 047P3*, 047Q3*, 047R3*, 047S3*, 047T3*, 047U3*, 047V3*, 047W3*, 047Y3*, 04C00ZZ, 04C03ZZ, 04CC0Z*, 04CC3Z*, 04CC4Z*, 04CD*, 04CH*, 04CJ*, 04CK*, 04CK*, 04CL*, 04CM*,

04CN*, 04CP*, 04CQ*, 04CR*, 04CS*, 04CT*, 04CU*, 04CV*, 04CY*, 04100J7, 04100J8, 04100JF, 04100JG, 04100JH, 04100JJ, 04100JK, 041C0JD, 041C0JH, 041D0JF, 041D0JJ, 041H0JH, 041H0KJ, 041H4JH, 041J0JJ, 041J4JJ, 041K0J*, 041K0Z*, 041L09*, 041L0J*, 041L0K*, 041L0Z*, 041M*, 041N*, 041S0JQ, 041U0AP, 0Y6*

CPT code: 37205, 37206, 37207, 37208, 37236, 37237, 37184, 37185, 37186, 35302, 35303, 35304, 35305, 35306, 35331, 35351, 35355, 35361, 35363, 35371, 35372, 35381, 35452, 35454, 35456, 35459, 35470, 35472, 35473, 35474, 35483, 35492, 35493, 35495, 35521, 35533, 35537, 35538, 35539, 35540, 35556, 35558, 35563, 35565, 35566, 35571, 35583, 35585, 35587, 35621, 35623, 35637, 35638, 35646, 35647, 35654, 35656, 35661, 35663, 35665, 35666, 35671, 35700, 35876, 35879, 35881, 35883, 35884, 37184, 37185, 37186, 37205, 37206, 37207, 37208, 0236T, 0237T, 0238T, 37225, 37224, 37227, 37226, 37222, 37223, 37220, 37221, 37229, 37228, 37231, 37230, 37233, 37232, 37235, 37234, 35548, 27590, 27591 ,27592, 27598, 27888, 27889, 28800, 28805

S2 Text: STARR-OMOP CONCEPT IDs for MACCE and AE

Concept IDs used for Stroke:

444091,374371,37395562,35610084,35610085,35610099,4006294,4006976,4009154,46270031,43530687,43530688,43531617,44782427,443864,43530679,42539254,42535100,4058289,42535227,37109056,37309663,43530702,43530703,439276,376094,4100252,374326,4100231,437308,4110192,4108356,376714,4108359,4110194,4108360,4110195,4111715,4111716,4111717,4110197,434056,4112026,4109870,372924,

4046089,4047747,4046090,4046237,381316,4045734,4043731,4045735,4046360,4045742,4043734,4048785,4046444,4338905,43531609,3188164,3185378,443790,43531621,373503,407708,4078315,761994,4116269,374384,4102928,37018688,374060,42535411,42539256,4164092,46270381,43531622,4102124,4119140,4121341,4141405,4146185,4143274,4207618,42539787,42535510,

762343,43530732,4263370,437540,4222582,4237180,3174555,4131383,4144150,4139517,4142739,762627,762629,443605,4196146,762755,443454,4198930,762933,762935,763014,765664, 40479572,40479575,40480002,40480449,40480475,40480938,40481762,40481842,40484513, 40484522,40484120,40485430,40489292,764362,764445,764446,764447,764501,764502,76450,37208181,43530749,43021377,4171852,4171853,442263,379778,381591,374055,442615,4273526,4278243,4288310,44782934,44782551,45766108,45766110,45772786,45767658,377254,46272244,

37016888,441874,37109512,36717605,36717248,37110237,37110240,37116473,42536240,42536241,42537643,375557,4296331,4213731,3184775,3188677,4311124,43531583,4338227,4230479, 43531592,4319330,4317291,4317292,4319146,43531605,43531607,444091,374371,37395562,35610084,35610085,35610099,4006294,4006976,4009154,4009654,46270031,43530687,43530688,43531617,43020423,44782427,443864,43530679,43530683,761110,42535096,4253509

7,42539254,42535100,4058289,42535227,37109056,36716999,37309663,43530702,43530703,439276,376094, 4100252,374326,437308,439296,439295,4110189,4110190,4110192,4108356,376714,4108359,4110194,4108360,4110195,4111715,4112023,4111716,4111717,4112024,4110197,434056,4112026,372924,765404,381316,4045734,4043731,4045735,4046360,4043734,4048785,4043737,4046444,4338905,43531609,3188164,3185378,443790,43531621,4096157,443239,313226,373503,4077086,

761994,3174271,43020496,43020497,4116269,374384,4102928,374060,42535411,42539256,4164092,46270381,43531622,4102124,4119140,4121341,4141405,4146185,4143274,4207618,4194719,3176312,42539787,42535510,762343,42535511,43530732,46273649,44782773,434656,4148535,4263370,4159140,437540,4237180,4237181,3174555,4131383,4137209,4144150,4139517,443605,762700,765495,762707,765774,762708,765775,4196146,762755,443454,4198930,762926,762933,762934,762935,765907,762936,762951,762954,763014,765664,763267,40479572,40479575,40480002,40480449,40480475,40480938,40481762,40481842,40484513,40484522,40484120,40485430,40489292,764362,764445,764446,764447,764496,764497,764499,764500,764501,764502,

764504,37208181,43530749,43021930,43021377,4171853,442263,316437,379778,374055,442615,4273526,4274969,4278243,764819,42535685,765694,4288310,44782934,44782551,45765600,45766070,45766073,45766088,45771319,45766108,45766110,45772786,45767657,45767658,377254,46272244,37016888,441874,37109512,36717605,36717248,37110237,37110238,37119077,37110678,381036,42539416,42539417,3169251,375557,42538980,42538826,4296331,437847,4213731,3184775,3188677,4311124,433195,4338227,4230479,43531592,4319330,4317291,4317292,43531605,43531607,4035152,4020946,4050288,2107740,2108350,2108351,4137380,4283095,4311546,4047016,2108328,42627938,2000059,2100954,2100955,4050288,4261515,

2108350,2108351,2002027,4137380,2853681,2728093,43019013,42895767,43019368,42895769,43018431,42895771,2853682,2728094,43018432,42895774,43020109,42895776,43019106,42895778,2849000,2728095,2808381,2728096,43019281,42895861,43019208,42895863,43018772,42895865,2888451,2728097,43019369,42895868,43018773,42895870,43019698,42895872,2836524,2728098,2888452,2728099,43018774,42895876,43019926,42895878,43018922,42895880,2800421,2728100,43018923,42895883,43019525,42895885,43019370,42895887,2794945,2728101,2820838,2728102,43018433,42895891,43018499,42895893,43018775,42895895,2849001,2728103,43019613,42895950,43019526,42895952,43018776,42895954,2828661,2728104,2841231,2728105,43018348,

42895958,43018924,42895960,43018925,42895962,2794946,2728106,43019699,42895965,43019371,42895967,43019815,42895969,2794947,2728107,2893522,2728108,43018500,42895973,43019614,42895975,43018501,42895977,2893523,2728109,43019816,42895980,43019700,42895982,43018614,42895984,2853683,2728110,2828662,2728111,43018926,42896026,43019209,42896028,43019210,42896030,2888453,2728112,43019211,42896033,43019107,42896035,43018927,42896037,2800423,2728113,2820842,2728114,43020110,42896041,43019212,42896043,43019372,42896045,2820843,2728115,43018502,42896048,43018503,42896050,43019213,42896052,2820844,2728116,2849116,2728117,43019373,42896056,43018928,42896058,43019615,42896097,2820845,2728118,43019817,42896100,43018777,42896102,43019282,42896104,2836525,2728119,2841232,2728120,43019014,42896108,43018505,42896110,43019214,42896112,

2813443,2728121,43019928,42896115,43019374,42896117,43019108,42896119,2853685,2728
122,2800424,2728123,43018778,42896123,43018779,42896125,43018615,42896127,2849117,2
728124,43019283,42896130,43019375,42896132,43019616,42896172,2808383,2728125,28412
33,2728126,43019376,42896176,43018506,42896178,43019284,42896180,2836527,2728127,43
018780,42896183,43019701,42896185,43018781,42896187,2862344,2728128,2795098,272812
9,43019617,42896191,43019818,42896193,43019527,42896195,2813444,2728130,43019377,42
896198,43019819,42896200,43018507,42896202,2888455,2728131,2795099,2728132,4301961
8,42896206,43019619,42896246,43018782,42896248,2853686,2728133,43019620,42896251,43
019215,42896253,43019216,42896255,2893525,2728134,2808386,2728135,43018508,4289625
9,43019621,42896261,43020111,42896263,2813445,2728136,43018929,42896266,43019378,42
896268,43019379,42896270,2853687,2728137,2808387,2728138,43019217,42896274,4301970
2,42896276,43019218,42896278,2849118,2728139,43019929,42895007,43018509,42895009,43
019528,

42895011,2875249,2728140,2836528,2728141,43019380,42895015,43019930,42895017,43018
930,42895019,2841234,2728142,43020112,42895022,43019381,42895024,43019703,42895026,
2841235,2728143,2808388,2728144,43020113,42895030,43020114,42895032,43020115,42895
034,2808389,2728145,43019015,42895037,43018349,42895039,43019820,42895041,2841236,
2728146,2849282,2728404,2800906,1531956,2728405,2795307,2728406,2800907,2728407,289
3686,1531955,2728408,2841401,2728409,2849283,2728410,2836699,1531954,2728411,
2853856,2728412,2813609,2728413,2813610,1531953,2728538,2853857,2728539,2826241,272
8540,2893688,1531952,2728541,2836700,2728542,2800909,2728543,2862509,1531951,
2728544,2836701,2728545,2888633,2728546,2893689,1531950,2728547,2875415,2728548,287
5416,2728549,2867382,1531949,2728550,2841404,2728551,2867383,2728552,2867384,153194
8,2728553,2862510,2728554,4019038,4049536,4049538,4050288,4144865,4226813,4178631,4
283095

Concept IDs for cardiovascular events:

37016102,35610091,35610093,46269996,36712779,35611570,35611571,37016181,46270159,4
6274044,36712982,36712983,36712984,37309713,35615053,36712985,4051874,4058723,3720
9632,37309630,44782769,314666,439693,4108669,4108670,321318,4111393,4108673,4108678
,438172,4108679,4108219,4108680,4020601,4068938,4329847,44782712,4124682,4119942,41
19455,4119457,4119944,4121464,4119945,4121465,4119946,4124684,4121466,4119947,31903
9,4119949,4121467,4119950,4121468,4119951,4119953,4124687,4136419,4151046,43021858,
4116486,4147223,4126801,4145721,4146343,4199962,4138833,4155007,4155962,4161973,415
5963,4161974,4161456,4161457,4155008,4155009,765132,4296653,4270024,4184827,319844,
315286,4185302,4187067,4185932,4168972,4139345,4175846,42537536,438170,4303359,4353
1588,37312532,4310270,315830,4231426,3180023,4242670,4011931,4006788,

4336464,4337056,4336465,4339629,4337737,4336466,4336467,4337741,4081578,2107216,210
7217,2107218,2107219,2107220,2107221,2107222,2107223,2107224,2107226,2107227,210722
8,2107231,2107242,2107243,2107244,4283892,4309432,4253805,4305509,4225903,4339288,4
3527994,43527995,43527996,43527997,43527998,43527999,43527908,43527909,43528000,43
528001,43528002,43528003,43528004,2313795,2313796,2313801,2313802,2313803,2313804,2

313810,2313811,42894241,42894242,42894243,42894244,42894245,42894246,2724714,27247
15,2724716,2724717,2724718,2724719,2724720,2724721,2724722,2724723,2724724,2724725,
2724726,2724727,2724728,2724729,2724730,2724731,2724732,2724733,2724734,2724735,272
4736,2724737,2724738,2724739,2724740,2724741,2724742,2724743,2724744,2724745,428942
47,42894248,42894249,42894250,42894251,42894252,2724746,2724747,2724748,2724749,272
4750,2724751,2724752,2724753,2724754,2724755,2724756,2724757,2724758,2724759,272476
0,2724761,2724762,2724763,2724764,2724765,2724766,2725213,2725214,2725215,2725216,2
725217,2725218,2725219,2725220,2725221,42894253,42894254,42894255,42894256,4289425
7,42894258,2725222,2725223,2725224,2725225,2725226,2725227,2725228,2725229,2725230,

2725231,2725232,2725233,2725234,2725235,2725236,2725237,2725238,2725239,2725240,272
5241,2725242,2725243,2725244,2725245,2725246,2725247,2725248,2725249,2725250,272525
1,2725252,2725253,42894223,42894224,42894225,42894226,42894227,42894228,2725254,272
5255,2725256,2725257,2725258,2725259,2725260,2725261,2725262,2725263,2725264,272526
5,2725266,2725267,2725268,2725269,2725270,2725271,2725272,2725273,2725274,2725275,2
725276,2725277,2725278,2725279,2725280,2725281,2725282,2725283,42894229,42894230,42
894231,42894232,42894233,42894234,2725284,2725285,2725286,2725287,2725288,2725289,2
725290,2725291,2725292,2725293,2725294,2725295,2725296,2725297,2725298,2725299,2725
300,2725301,2725302,2725303,2725304,2725305,2725306,2725307,2725308,2725309,2725310
,2725311,2725312,2725313,2725314,2725315,42894235,42894236,42894237,42894238,

42894239,42894240,2725316,2725317,2725318,2725319,2725320,2725321,2725322,2725323,2
725324,2725325,2725326,2725327,2725328,2725329,2725330,2725331,2725332,2725333,
2725334,2725335,2725336,2725337,2725338,2725339,2725340,2725341,2725342,2725343,272
5344,2725345,42894398,42894399,42894400,42894401,42894402,42894403,2725346,2725347,
2725348,2725349,2725350,2725351,2725352,2725353,2725354,2725355,2725356,2725357,272
5358,2725359,2725360,2725361,2725362,2725363,2725364,2725365,2725366,2725367,272536
8,2725369,2725370,2725371,2725372,2725373,2725374,2725375,2725376,2725377,42894404,
42894405,42894406,42894407,42894408,42894409,2725378,2725379,2725380,2725381,27253
82,2725383,2725384,2725385,2725386,2725387,2725388,2725389,2725390,2725391,2725392,
2725393,2725394,2725395,2725396,2725397,2725398,2725399,2725400,2725401,2725402,272
5403,2725404,2725405,2725406,2725407,2725896,2725897,42894467,42894468,42894469,428
94470,42894471,42894472,2725898,2725899,42894473,42894474,42894475,42894476,428944
77,42894478,2725900,2725901,2725902,2725903,2725904,2725905,42894479,42894480,42894
481,42894482,42894483,42894484,2725906,2725907,42894667,42894668,42894669,

42894670,42894671,42894672,2725908,2725909,2725910,2725911,2725920,2725921,4289468
5,42894686,42894687,42894688,42894689,42894690,2725922,2725923,42894691,42894692,
42894693,42894694,42894695,42894696,2725924,2725925,2725926,2725927,2725928,272592
9,42894697,42894698,42894699,42894700,42894701,42894702,2725930,2725931,42894703,

42894704,42894705,42894706,42894707,42894708,2725932,2725933,2725934,2725935,27259
44,2725945,42894586,42894587,42894588,42894589,42894590,42894591,2725946,2725947,42
894592,42894593,42894594,42894595,42894596,42894597,2725948,2725949,2725950,272595
1,2725952,2725953,42894598,42894599,42894600,42894601,42894602,42894603,2725954,272
5955,42894604,42894605,42894606,42894607,42894608,42894609,2725956,2725957,2725958,

2725959,2725968,2725969,42894622,42894623,42894624,42894625,42894626,42894627,2725970,2725464,42894628,42894629,42894630,42894631,42894632,42894633,2725465,2725466,2725467,2725468,2725469,2725470,42894643,42894644,42894645,42894646,42894647,42894648,2725471,2725472,42894649,42894650,42894651,42894652,42894653,42894654,2725473,2725474,2725475,2725476,4008625,46269996,4006788,3170433,4000732,4031850,

4031996,3190422,4020653,44789455,4337056,4336465,3173558,3180695,4106548,4233420,2001500,2001501,2001504,2001506,2001509,2001516,2001517,2001518,4283892,4161974,4161456,4298487,4264285,4219321,4233485,2108631,4184832,4171077,4177223,4178148,4178622,4329263,4181025,4167173,43531439,43531440,4284104,37111313,4328103,35607959,4217445,4225903,4231998,4234990,43527994,43527995,43527996,43527997,43527998,43527999,43527908,43527909,2313801,2313802,2313803,2313810,2313811,43018564,42894241,42894242,42894243,42894244,42894245,42894246,2874560,2724714,2724715,2724716,2724717,2724718,2724719,2822626,2724720,2724721,2724722,2724723,2724724,2724725,2794161,2724726,2724727,2724728,2724729,2724730,2724731,2874561,2724732,2724733,2724734,2724735,2724736,2724737,2874562,2724738,2724739,2724740,2724741,2724742,2840415,2724743

2887768,2724744,2840416,2724745,43018392,42894247,42894248,42894249,42894250,42894251,42894252,2874563,2724746,2724747,2724748,2724749,2724750,2724751,2815230,2724752,2724753,2724754,2724755,2724756,2724757,2807538,2724758,2827849,2724759,2724760,2724761,2724762,2724763,2724764,2887769,2724765,2724766,2725213,2725214,2725215,2725216,2848163,2725217,2725218,2725219,2725220,2725221,43019880,42894253,42894254,42894255,42894256,42894257,42894258,2848164,2725222,2725223,2725224,2725225,2725226,2725227,2861498,2725228,2725229,2725230,2725231,2725232,2725233,2892836,2725234,2725235,2725236,2725237,2725238,2725239,2815231,2725240,2725241,2725242,2725243,2725244,2725245,2835845,2725246,2725247,2725248,2725249,2725250,2840417,2725251,2887770,2725252,2840418,2725253,43019881,42894223,42894224,42894225,42894226,42894227,42894228,2848165,2725254,2725255,2725256,2725257,2725258,2725259,2866375,2725260,2725261,2725262,2725263,2725264,2725265,2840419,2725266,2879793,2725267,2725268,2725269,2725270,2725271,2725272,2848166,2725273,2725274,2725275,2725276,2725277,2725278,2840420,2725279,2725280,2725281,2725282,2725283,43020067,42894229,42894230,42894231,42894232,42894233,42894234,2892837,2725284,2725285,2725286,2725287,2725288,2725289,2879794,2725290,2725291,2725292,2725293,2725294,2725295,2848167,2725296,2725297,2725298,2725299,2725300,2725301,2874566,2725302,2725303,2725304,2725305,2725306,2725307,2848168,2725308,2725309,2725310,2725311,2725312,2799778,2725313,2840421,2725314,2794162,2725315,43018666,

42894235,42894236,42894237,42894238,42894239,42894240,2815232,2725316,2725317,2725318,2725319,2725320,2725321,2887772,2725322,2725323,2725324,2725325,2725326,2725327,2822627,2725328,2815233,2725329,2725330,2725331,2725332,2725333,2725334,2861499,2725335,2725336,2725337,2725338,2725339,2725340,2866376,2725341,2725342,2725343,2725344,2725345,43018728,42894398,42894399,42894400,42894401,42894402,42894403,2866377,2725346,2725347,2725348,2725349,2725350,2725351,2840422,2725352,2725353,2725354,2725355,2725356,2725357,2815234,2725358,2725359,2725360,2725361,2725362,2725363,2807540,2725364,2725365,2725366,2725367,2725368,2725369,2874567,2725370,2725371,2725372,2725373,2725374,2807541,2725375,2887773,2725376,2848169,2725377,43018982,42894404,428

94405,42894406,42894407,42894408,42894409,2879795,2725378,2725379,2725380,2725381,2725382,2725383,2835847,2725384,2725385,2725386,2725387,2725388,2725389,2848170,2725390,2799779,2725391,2725392,2725393,2725394,2725395,2725396,2794164,2725397,2725398,2725399,2725400,2725401,2725402,2874568,2725403,2725404,2725405,

2725406,2725407,2892846,2725896,2725897,43019489,42894467,42894468,43019490,42894469,42894470,43018399,42894471,42894472,2822645,2725898,2725899,43018883,42894473,42894474,43019334,42894475,42894476,43018679,42894477,42894478,2861520,2725900,2725901,2822646,2725902,2725903,2807561,2725904,2725905,43019181,42894479,42894480,43018884,42894481,42894482,43020079,42894483,42894484,2815243,2725906,2725907,43019454,42894667,42894668,43018400,42894669,42894670,43019994,42894671,42894672,2799912,2725908,2725909,2815244,2725910,2725911,2879941,2725912,2725913,43018734,42894673,42894674,43018401,42894675,42894676,43019182,42894677,42894678,2852854,2725914,2725915,43019183,42894679,42894680,43019995,42894681,42894682,43020080,42894683,42894684,2807562,2725916,2725917,2887792,2725918,2725919,2835860,2725920,2725921,43018680,42894685,42894686,43018885,42894687,42894688,43018735,42894689,42894690,2879943,2725922,2725923,43018568,

42894691,42894692,43018681,42894693,42894694,43019072,42894695,42894696,2822648,2725924,2725925,2799913,2725926,2725927,2822649,2725928,2725929,43019184,42894697,42894698,43019767,42894699,42894700,43018886,42894701,42894702,2815245,2725930,2725931,43019885,42894703,42894704,43019455,42894705,42894706,43020081,42894707,42894708,2827866,2725932,2725933,2827986,2725934,2725935,2835861,2725936,2725937,43019185,42894709,42894710,43018569,42894711,42894712,43018737,42894713,42894714,2822650,2725938,2725939,43018887,42894715,42894716,43019589,42894717,42894718,43018888,42894584,42894585,2822651,2725940,2725941,2879944,2725942,2725943,2848318,2725944,2725945,43019186,42894586,42894587,43019768,42894588,42894589,43018738,42894590,42894591,2887793,2725946,2725947,43019889,42894592,42894593,43019890,42894594,42894595,43018570,42894596,42894597,2879945,2725948,2725949,2848319,2725950,2725951,2835862,2725952,2725953,43019590,42894598,42894599,43018986,42894600,42894601,43019769,42894602,42894603,2852856,2725954,2725955,43018682,42894604,42894605,43019335,42894606,42894607,43018683,42894608,42894609,2852857,2725956,2725957,2874583,2725958,2725959,2861654,2725960,2725961,43019491,42894610,42894611,43020082,42894612,42894613,43019891,42894614,42894615,2879946,2725962,2725963,43019456,42894616,42894617,43019770,42894618,42894619,43018987,42894620,42894621,2887794,2725964,2725965,2827989,2725966,2725967,2815246,2725968,2725969,43019591,42894622,42894623,43019892,42894624,42894625,43019457,42894626,42894627,2866520,2725970,2725464,43018739,42894628,42894629,43019336,42894630,42894631,43018684,

42894632,42894633,2874585,2725465,2725466,2852858,2725467,2725468,2848321,2725469,2725470,43019187,42894643,42894644,43018740,42894645,42894646,43020083,42894647,42894648,2835863,2725471,2725472,43019458,42894649,42894650,43019886,42894651,42894652,43018402,42894653,42894654,2835864,2725473,2725474,2815247,2725475,2725476,2822652,2725477,2725478,43019893,42894655,42894656,43019459,42894657,42894658,43019073,42894659,42894660,2807563,2725479,2725480,43018889,42894661,42894662,43018741,42894663,42894664,43018571,42894665,42894666,2887795,2725481,2725482,2852859,2725483,2725

484,2794352,42894738,2725621,2799942,42894739,2725622,2866541,42894740,2725623,2879969,42894741,2725624,2799943,42894742,2725625,2879971,42894743,2725626,2794353,42894744,2725627,2879972,42894745,2725628,2815386,42894746,2725629,2840573,42894747,2725630,2892984,42894748,2725631,2815387,42894749,2725632,4006788,4000732,4031850,4063237,4020213,4309432,2833693,2787189,2805718,2787192,2893432,2787194,2880403,2787197,4233485,4184832,4216130,4180760,4284104,42537730,4305509,4231998

Concept IDs used for amputation:

2784242,2784243,2784244,2784245,2784246,2784247,2784248,2784250,2784251,2784474,2784475,2784476,2784477,2784478,2784479,2784480,2784481,2784482,2784483,2784484,2784485,2784486,2784487,2784488,2784489,2784490,2784491,2784492,2784493,2784494,2784495,2784496,2784497,2784498,2784499,2784500,2784501,2784502,2784503,2784504,2784505,2784506,2784507,2784508,2784509,2784510,2784511,2784512,2784513,760545,760563,4002166,4054498,4058850,3173821,4108565,4078563,3168091,4054983,4143795,4169822,2105209,2105210,2105211,2105222,2105223,4106053,4119910,2105806,4183102,4177620,4272232,4159766,4218050,4219032,4264289,4217266,4266202,4179666,43531147,46272657,36717437,36715395,37118455,37115743,37116276,37204044,4302020,4195136,2006242,2006243,4338257

S1 Table: NRI RESULTS FOR PAD DETECTION MODELS

```
----------------------------------------
     Reclassification table
 ----------------------------------------
```

Updated Model

**Initial Model**	**Low Risk (<5%)**	**Intermediate Risk (5-25%)**	**High Risk (>25%)**	**Percent Reclassified**
**Low Risk (0-5%)**	99	9	0	8%
**Intermediate Risk (5-25%)**	14	83	2	16%
**High Risk (>25%)**	0	2	69	3%

NRI(Categorical) [95% CI]: 0.0694 [0.0016 - 0.1372] ; p-value: 0.04471
NRI(Continuous) [95% CI]: -0.1433 [-0.412 - 0.1254] ; p-value: 0.29591
IDI [95% CI]: 0.0092 [0.0041 - 0.0143] ; p-value: 0.00046

S2 Table: METRICS OF COMBINED PRS PAD DETECTION MODEL

Sensitivity	0.65
PPV	0.85
Specificity	0.97
NPV	0.92

S3 Table: NRI RESULTS FOR AE PREDICTION MODEL

```
 _________________________________________

     Reclassification table
 _________________________________________
```

Updated Model

**Initial Model**	**Low Risk (<10%)**	**Intermediate Risk (10-20%)**	**High Risk (>20%)**	**Percent Reclassified**
**Low Risk (0-10%)**	1	2	0	67%
**Intermediate Risk (10-20%)**	0	11	1	8%
**High Risk (>20%)**	0	0	69	0%

NRI(Categorical) [95% CI]: -0.0221 [-0.1008 - 0.0566] ; p-value: 0.58162
NRI(Continuous) [95% CI]: -0.2779 [-0.696 - 0.1402] ; p-value: 0.19261
IDI [95% CI]: -0.0103 [-0.0243 - 0.0036] ; p-value: 0.1474
